# Evaluating Antimicrobial Activity and Wound Healing Effect of Rod-Shaped Nanoparticles

**DOI:** 10.3390/polym14132637

**Published:** 2022-06-28

**Authors:** Wafaa E. Soliman, Heba S. Elsewedy, Nancy S. Younis, Pottathil Shinu, Lamis E. Elsawy, Heba A. Ramadan

**Affiliations:** 1 Department of Biomedical Sciences, College of Clinical Pharmacy, King Faisal University, Alhofuf 36362, Al-Ahsa, Saudi Arabia; spottathail@kfu.edu.sa; 2 Department of Microbiology and Immunology, Faculty of Pharmacy, Delta University for Science and Technology, Mansoura 11152, Egypt; lamis.elbaz@deltauniv.edu.sa (L.E.E.); heba.musa@deltauniv.edu.eg (H.A.R.); 3 Department of Pharmaceutical Sciences, College of Clinical Pharmacy, King Faisal University, Alhofuf 36362, Al-Ahsa, Saudi Arabia; helsewedy@kfu.edu.sa (H.S.E.); nyounis@kfu.edu.sa (N.S.Y.)

**Keywords:** gold nanoparticles, characterization, antibacterial, antifungal, diabetic, wound healing

## Abstract

Presently, the nanotechnology approach has gained a great concern in the media of drug delivery. Gold nanoparticles (Au-NPs) specially having a non-spherical structure, such as gold nanorods (GNR), are attracting much interest as antibacterial agent and many other medical fields. The aim of the current investigation was to characterize Au-NPs and investigate their antimicrobial and wound healing efficacy in diabetic animals. Material and methods: Au-NPs were characterized using a UV-Vis spectrophotometer, estimating their particle size, polydispersity (PDI), and assessing their morphological characters. Further, Au-NPs were estimated for their antibacterial and antifungal behavior. Ultimately, in vivo activity of Au-NPs was evaluated against excision wound healing in STZ-induced diabetic animals. Results: Au-NPs were found to show maximum absorption at 520 nm. They exhibited a particle size of 82.57 nm with a PDI value of 0.323. Additionally, they exhibited good antimicrobial activity against different bacterial strains. Topical application of Au-NPs caused a significantly increased percentage of wound area reduction, lesser time needed for epithelialization, and augmented hydroxyproline, collagen, and hexosamine levels demonstrating enhanced healing processes. Furthermore, Au-NPs displayed a significant intensification in angiogenesis-related factors (HIF-1α, TGF-β1, and VEGF), and antioxidant enzymes activities (CAT, SOD, GPx) as well as mitigated inflammatory mediators IL-6, IL-1β, TNF-α, and NF-κB) and lipid peroxidation (MDA). Conclusion: Au-NPs exhibited proper particle size, and rod-shaped particles, with efficient antimicrobial behavior against different bacterial strains. Furthermore, Au-NPs demonstrated a promising wound healing activity in STZ-induced diabetic animals.

## 1. Introduction

Bacterial infection is one of humanity’s most critical hazards. Bacteria can cause a variety of infections [1]. Gram-negative bacteria, such as *E. coli*, can cause infections in the urinary tract, gastrointestinal tract, and lungs. Gram-positive bacteria have the potential to harm the skin and other sensitive tissues. Existing antibiotics are no longer effective in controlling bacterial infection due to the overuse of classical, small organic molecule-based antibiotics [2], which resulted in multiple global disasters as a result of the widespread of resistant fungus and bacteria [3]. The challenge of antibiotic-resistant microorganisms, as well as the focus on healthcare expenditures, drives researchers to come up with pioneering ways to generate powerful antimicrobial drugs to combat bacterial resistance and lower costs [4].

Nanotechnology, a new discipline of research, has a wide range of applications in fields such as agriculture, electronics, and medicine. NPs and their conjugates have been extensively explored in antibacterial therapy [5]. Antibacterial, antimalarial, and antibiofilm agents have all been utilized with gold NPs (Au-NPs). Au-NPs have a wide range of biological uses, including medicinal and gene therapy, as well as diagnostic biosensors. Au-NPs could be synthesized using various methods including coprecipitation, chemical reduction, seeding, and hydrothermal method [6]. NPs are simple to be synthesized using a co-precipitation method especially for preparing iron oxide NPs [7,8]. On the other hand, the chemical reduction method was widely used for preparing NPs such as silver and gold NPs [9]. It is an effective method by which the aqueous salt of the metal is reduced to produce the NPs. Further, the obtained NPs are stabilized with certain stabilizers, mainly; cetyltrimethylammonium bromide (CTAB) for gold NPs and sodium citrate for silver ones [10]. Regarding seeding method, the NPs were developed by reduction of salts in the aqueous media and controlling the size of the obtained NPs by the stabilizing agent [11]. However, hydrothermal method depends on certain reaction under high temperature and pressure for growing NP crystals [12].

Gold nanorods (GNR), having a non-spherical structure, are attracting a lot of interest as antibacterial candidates. They extensively absorb near-infrared light that is within an acceptable wavelength window for therapeutic applications [13], resulting in local hyperthermia that can be exploited to eradicate germs, in addition to their biocompatibility and simplicity of functionalization [14]. Additionally, it was reported that the antibacterial activity of NPs came from their reaction and deactivation for the bacterial enzymes [15]. It was previously investigated that the antibacterial activity of Au-NPs prepared with CTAB exhibited a toxic influence against the bacterial culture [16]. Furthermore, the antifungal activity of GNR against Candida strains was studied and confirmed earlier [17]. Two types of NPs are broadly used for wound healing, one of them acts as a route of delivering the active agent while the other has its own properties that enable the wound healing action [18]. The later NPs such as metallic NPs that gained a great concern in the medical application including the enhancement of wound healing [19].

Wound healing is the body’s natural reaction to fix and rebuild damaged skin tissues. It is the most prevalent occurrence in human life as a result of traumatic, surgical, or burn traumas, as well as chronic disorders. A significant risk of bacterial infection is associated with poor wound healing [20]. Diabetes is linked to poor wound healing due to abnormal cellular and cytokine responses, infection, poor vascularization, and neuropathy. Recent promising treatment techniques for speeding diabetic wound healing include nanotherapeutics-based compounds designed within 1–100 nm levels, which include NPs [21]. Different nanoformulations exerted a great influence in the field of wound healing especially in diabetic patients [22]. Interestingly, the antimicrobial and antioxidative effects of gold NPs proved very effective in wound healing and regeneration of damaged collagen tissues [23]. Another study reported that topical Au-NP application with epigallocatechin gallate and α-lipoic acid significantly accelerated mouse cutaneous wound healing through anti-inflammatory and anti-oxidant effects [24]. Wound healing efficiency of GNR was formerly investigated by Mahmoud et al., where the prepared nanorods exhibited strong antibacterial activity against the most common skin pathogens and facilitated wound healing [25]. Moreover, Hassan et al investigated the potential of the wound healing mechanism of the developed nanorods [26]. Furthermore, it was reported that the hydrogel of the developed GNR revealed a significant wound healing properties following topical application to wounds [27].

As a result, the goal of the current research is to describe gold rod-shaped NPs, incorporated into gel formulation for topical application and explore their antibacterial and wound-healing effect in diabetic wounded animals treated with Au-NPs.

## 2. Materials and Methods

### 2.1. Materials

Tetrachloroauric acid, cetyltrimethyl ammonium bromide, ascorbic acid, sodium borohydride (NaBH_4_), silver nitrate, and Hydroxypropyl methylcellulose (HPMC) were bought from Sigma-Aldrich Co. (St Louis, MO, USA). All other chemicals were of analytical grade.

### 2.2. Synthesis of Au-NPs

Au-NPs development was conducted by simply preparing seed and growth solution. For the seed solution, 5 mL, 0.2 M of cetyltrimethyl ammonium bromide (CTAB) was stirred with equivalent volume of 0.0005 M HAuCl4. Next, 0.6 mL of ice cold 0.01 M NaBH_4_ was added, which resulted in forming the brownish-yellow colour seed solution. Stirring was kept for 2 min and then stored at 25 °C. Regarding growth solution, cetyltrimethyl ammonium bromide (5 mL, 0.2 M) was added to (0.05 mL) of 0.004 M AgNO_3_ solution and gently mixed with 5 mL of 0.0010 M HAuCl4. Next, 70 μL of 0.0788 M ascorbic acid as a mild reducing agent was added. The colour was changed from dark yellow to colorless. 12 µL of seed solution was mixed with growth solution at 30 °C after which the color was gradually changed [28]. 

### 2.3. Characterization of Synthesized Au-NPs

#### 2.3.1. UV–Visible Spectroscopic Determination

Estimating the UV-Vis spectroscopy for analyzing the surface plasmon resonance is very essential in the characterization of the formulated NPs. This estimation was carried out using a UV-Vis spectrometer (U.V. Spectrophotometer, JENWAY 6305, Stone, UK) in the wave length range of 200–800 nm [29].

#### 2.3.2. Dynamic Light Scattering Analysis and Zeta Potential

The particle size of the fabricated NPs together with its size distribution (PDI) was determined depending on the dynamic light scattering analysis technique. Additionally, the NPs zeta potential was estimated by determining the surface charges relying on the electrophoretic mobility. The evaluations were conducted using Zetasizer apparatus (Malvern Instruments Ltd., Worcestershire, UK) at 25 °C [30]. 

#### 2.3.3. Transmission Electron Microscopy (TEM)

The morphology of the synthesized Au-NPs was inspected via Transmission electron microscopy (TEM, JEM, 2100, Tokyo, Japan). Sample was prepared by adding one drop of the formulation over a carbon-coated copper grid and kept to dry using infrared lamp, then investigated at accelerating voltage 200kv.

### 2.4. Fourier Transform Infrared Spectroscopy (FTIR)

FTIR (FTIR spectrophotometer, SHIMADZU, IRAFFINITY-1S, Japan, Kyoto, Japan) was employed for identifying the functional group in the fabricated NP and for detecting any drug excipient interaction. The study was done using KBr pellet method, where the NP sample was placed over a KBr plate and allowed to be dried at vacuum. The spectra of FTIR were recorded among 4000 and 400 cm^−1^ [31].

### 2.5. Fabrication of Topical Au-NPs Gel 

Topical formulation integrating Au-NP was prepared in order to be easier and more efficient for application over skin. Simply, 4% *w*/*w* gelling agent (HPMC) was sprinkled over distilled water and kept stirring on a magnetic stirrer (Jeio Tech TM-14SB, Medline Scientific, Oxfordshire, UK) till getting smooth hydrogel base. 250 µL of the developed Au-NP was mixed with them and vortexed for 5 min till obtaining hydrogel containing Au-NP [32].

### 2.6. Characterization of the Developed Au-NPs Gel Preparation

#### 2.6.1. Visual Examination

The developed Au-NP gel was visually examined for its physical appearance, homogeneity, and color. 

#### 2.6.2. pH Measurement

To ensure the safety of the developed gel and avoid the possibility of irritation upon its application, the pH of the gel should be measured. Standardized pH meter (MW802, Milwaukee Instruments, Szeged, Hungary) was utilized for such measurement [33].

#### 2.6.3. Viscosity Measurement

Viscosity is a very essential parameter for topical formulations to be evaluated since improper viscosity would be inefficient and may cause discomfort for the patient [34]. Therefore, Brookfield viscometer (DV-II+ Pro, Middleboro, MA, USA) using spindle 63 was used to determine the viscosity of Au-NP gel formulation at 25 °C [35].

#### 2.6.4. Spreadability Determination

Same like, spreadability is another vital parameter to be calculated. It depends mainly upon the viscosity of the formulation. Proper spreadability help in the even and smooth application of the preparation over the affected area. Briefly, a sample of the gel was added in between two glass slides. Certain load (500 g) was fixed over them for 1 min. The spreadability is calculated by measuring the diameter of the formulation spreading area [36].

### 2.7. Antimicrobial Studies

#### 2.7.1. Culturing of the Microorganisms 

Both *Staph. aureus* (ATCC 10400) and *E. coli* (ATCC 25922) were used as Gram-positive and Gram-negative control organisms, respectively. However *Candida albicans* (ATCC 90028) was included as fungal control. All the microorganisms were obtained in freeze-dried form and rehydrated in Luria Bertani broth (10 g tryptone, 5 g yeast extract, 5 g NaCl l-1; Sigma–Aldrich, St. Louis, MO, USA) whereas *C. albicans*, which was cultured in Sabouraud dextrose broth (Sigma–Aldrich). Later the broth was solidified (by adding 1.5 percent agar) to preserve the viability of the microorganisms. These media was incubated at 37 °C (for bacteria) and 27 °C (for fungi), respectively, to enhance the growth of the microorganisms [37].

#### 2.7.2. The Antimicrobial Activity of Au-NPs 

For each experiment, a single colony of each organism was inoculated into 5 mL of 3% (wt%) tryptic soy broth (TSB) and growing it in a shaking incubator for overnight. The bacteria were diluted in 3% TSB so as to reach an optical density of 592 nm (OD592) = 0.52 that corresponds to 10^9^ bacteria/mL. The culture was diluted further in TSB before being plated at a concentration of 10^6^ bacteria/mL. AU-NPs at a concentration of 2 nmol/mL were diluted with TSB to 1, 0.5, 0.25, 0.125, till 0.008 nmol/mL solutions. Of these, 50 µL of diluted bacterial culture was added to each well of the 96-well plate except in negative control. Each treatment was replicated three times on each plate. After incubation at 37 °C for 24 h (for bacteria) and 48 h (for the fungus), 0.015% resazurin solution was added to all wells (20 µL per well) followed by incubation at 37 °C for 2–4 h and monitored for the change in check color.

#### 2.7.3. Minimum Bactericidal and Fungicidal Concentration Determination 

Following the MIC analysis, the minimum bactericidal concentration (MBC) and minimum fungicidal concentration (MFC) were determined. A total of fifty microliters of aliquots were taken from all tubes that exhibited no signs of growth or change in optical density at 600 nm. Aliquots were then seeded aseptically on nutrient agar plates and incubated at 37 °C for 1 day. The lowest concentration of the antimicrobial agent when 99.9% of the bacterial or fungal strains were completed was referred to as MBC and MFC endpoints, respectively [38].

#### 2.7.4. Scanning Electron Microscopy (SEM) Imaging of *S. aureus*, *E. coli* and *C. albicans* treated with Au-NPs

Using a poly-l-lysin-coated slide, SEM imaging of *S. aureus*, *E. coli*, and *C. albicans* treated with Au-NPs solution was performed. 100 μL of Au-NPs suspension (1.25 nM) were combined with 100 μL of Mueller–Hinton broth and 10 μL of microbe (about 1.5 × 106 CFU/mL) and incubated for 1 h. After centrifuging the mixture for 20 min at 15,000 rpm, the supernatant was removed, and the pellets were suspended in normal saline. After that, 50 μL of the suspension was poured onto the slide and allowed to dry. After that, the sample was fixed in 3% glutaraldehyde for 3 h and photographed using a scanning electron microscope. As a control, SEM imaging of untreated *S. aureus*, *E. coli* and *C. albicans* were carried out.

### 2.8. In Vivo Studies

#### 2.8.1. Animal’s Acquisition 

Male Wistar rats (200 ± 20 g, 6–8 weeks) were acquired from King Saud University, Riyadh, Kingdom of Saudi Arabia. Rats were maintained in the animal house under standard surroundings for a week prior to conducting the experiment. Rats were fed laboratory chow and water ad libitum. 

#### 2.8.2. Ethical Approval 

All experiments were appropriately executed in agreement with the “Ethical Conduct for Use of Animals in Research” Guidelines in King Faisal University. The Animal Research Ethics Committee at King Faisal University approved all animal care and experimental procedures with an ethical approval no (KFU-REC-2022- MAY -EA000632). 

#### 2.8.3. Induction of Diabetes 

Diabetes was induced in overnight-fasted rats via intraperitoneal (i.p.) injection of 65 mg/kg of a freshly prepared streptozotocin (STZ) (Sigma, St. Louis, MO, USA) dissolved in citrate buffer (pH 4.5). Rats were administered 10% glucose solution after 6 h of STZ injection for another 24 h, to deter the severe hypoglycemia triggered by the immense insulin release [39,40]. After three days of the STZ administration, fasting blood glucose level (BGL) was measured and rats with BGL higher than 200 mg/dL were considered diabetic and were used in the current study.

#### 2.8.4. Experimental Design

Excision wound models were used to evaluate the diabetic wound healing activity of Au-NPs in this investigation. Diabetic rats were arbitrarily separated into three groups in each model (*n* = 6). Group I: diabetic negative control, in which diabetic animals were granted to heal spontaneously without any treatment. Group II: diabetic positive control, in which diabetic rats were treated with the commercial standard ointment (Silver Sulfadiazine 1% ointment (SSD) (Dermazin®), manufactured by Medical Union Pharmaceuticals, Saudi Arabia). Group III: diabetic + Au-NPs (30 μg/kg), in which diabetic rats were treated with topical application of 30 μg/kg Au-NPs on wounds for once a day. Animals were checked for any wound contamination daily throughout the whole experiment. No antibiotic was given to any of the animals’ groups. At the end of the investigation and after the complete wound healing, the animals were sacrificed using 10 mg/kg of xylazine (Bayer, Leverkusen, Germany) and 25 mg/kg of ketamine (Pfizer Inc., New York, NY, USA) and wound tissue samples were collected and preserved in liquid nitrogen for further biochemical analysis. At the end of the experiment, blood samples were collected, centrifuged at 3000× *g* for 15 min, and the resulting serum was employed for subsequent biochemical assays. 

#### 2.8.5. Excision Wound Establishment 

Anesthetized rats were situated face down on the dissection pad. Before the wound creation, the dorsal region of the anesthetized animals was shaved, and the wound areas were cleaned via 70% ethanol. An open circular cut of 0.2 mm depth and 10 mm^2^ diameter was made on the back of each animal using a sterile scalpel. The animals were distributed randomly into the different groups as mentioned in the experimental design. Days of application of topical preparations started from day 0 to the day of complete wound healing.

#### 2.8.6. Measurement of Wound Area Parameters 

##### Macroscopic Investigation 

Wound photos were taken on days 0, 3, 7, 14, and 21 using a camera (Spot Insight QE; Diagnostic Instruments, Sterling Heights, MI, USA). The epithelialization time which is the number of days taken to drop off the dead tissue without any sign of raw wound [41]. The wound area was measured using a tracing paper by placing the paper on the wound on the 0th, 7th, 14th, and 21st day after excision wound establishment. The percentage of wound contraction was calculated as stated previously [42] using the following formula:

Wound contraction percentage = ((wound area day 0 − wound area day *n*)/wound area day 0) × 100 where *n* is the number of days until whole healing.

#### 2.8.7. Skin Tissues Measured Parameters 

The hydroxyproline content in wound tissues was measured as described earlier [43]. Concisely, the wound tissue samples obtained from different experimental animals were dried in a hot air oven at 60 °C to obtain similar weights and then hydrolyzed with 6 N hydrochloric acids (HCl) (1:10, w:v) at 130 °C for 4 h in sealed glass tubes. The attained hydrolysates were neutralized to pH 7.0 and subjected to oxidation via chloramine T. The reactions accomplished by the addition of perchloric acid (0.4 M), and Ehrlich reagent at 60 °C to develop the colors which were valued at 557 nm. A standard linear curve was performed, from which hydroxyproline concentrations were calculated and presented as μg/mg of dry tissue weight.

#### 2.8.8. Measurement of Angiogenesis Related Factors 

In the wound tissue homogenates, vascular endothelial growth factor (VEGF, Item No. LS-F5482), transforming growth factor-β1 (TGF-β1, Item No. LS-F24972) and hypoxia-inducible factor 1-alpha (HIF-1α, Item No. LS-F4225) levels were measured using the ELISA kit attained from LifeSpan BioSciences, Seattle, WA, USA.

#### 2.8.9. Measurement of Lipid Peroxidation and Antioxidant Enzymes Activities 

Lipid peroxidation was assessed using thiobarbituric acid reaction and stated as the malondialdehyde (MDA) level using an MDA kit (Cat. No. ab 118790, Abcam, Cambridge, MA, USA). Regarding the antioxidant activities, glutathione (GSH, Cat. No. 703002), glutathione peroxidase (GPx, Cat. No. 703102), superoxide dismutase (SOD, Cat. No. 706002) and catalase (CAT, Cat. No. 707002) were measured agreeing with the manufacturer’s procedures using the ELISA kits, (Cayman Chemicals, Ann Arbor, MI, USA).

As for the estimation of nitric oxide (NO), the stable end-products of nitric oxide (NO) biosynthesis were estimated by appraising the nitrite levels. Greiss reagent (500 μL; 1:1 solution of 1% sulphanilamide in 5% phosphoric acid and 0.1% napthaylamine diamine dihydrochloric acid in water) was placed with 100 μL of serum and absorbance was valued at 546 nm using spectrophotometer [44]. The nitrite concentration was calculated using a standard curve for sodium nitrite and expressed as nanograms per milligram of protein. 

#### 2.8.10. Measurement of the Inflammatory Cytokine 

Tumor Necrosis Factor-alpha (TNF-α, Cat. No. LS-F23150), Interleukin 8 (IL-8, Cat. No. LS-F9753) and Interleukin 1β levels by ELISA based kits obtained from LifeSpan Bio-Sciences, Seattle, WA, USA. The ELISA assays were implemented as per the manufacturer’s guidelines. 

### 2.9. Statistical Analysis 

Data are presented as the mean ± SE. Multiple comparisons were performed via one-way ANOVA, followed by Tukey’s test as a post hoc analysis, using a 0.05 level of probability as the significance level. All statistical analyses were achieved using GraphPad Prism (GraphPad Software Inc., San Diego, CA, USA,) software, version 8.

## 3. Results

### 3.1. Characterization of Au-NPs

#### 3.1.1. UV–Visible Spectroscopic Determination

**Figure 1:**
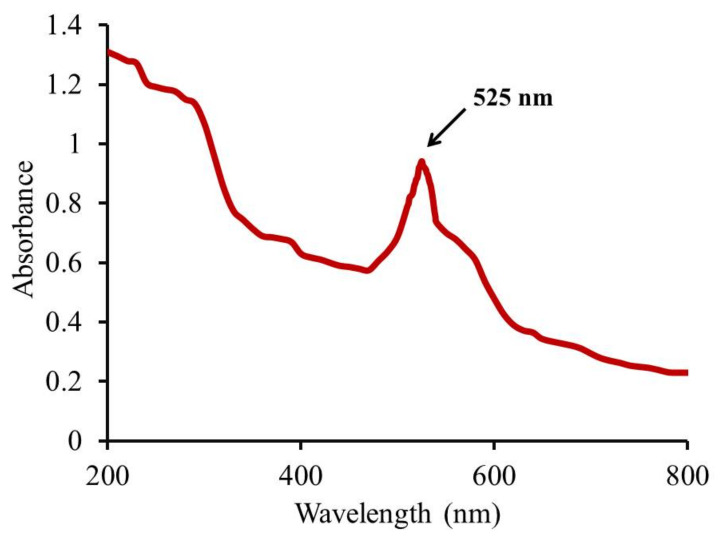
UV scanning of fabricated gold NP.

The spectrum of UV-visible spectroscopy of the fabricated Au-NPs was scanned between 200 and 800 nm and the result was shown in Figure 1. The band of the surface plasmon resonance appeared to be within 510–540 nm with a characteristic absorption peak at 525 nm. 

#### 3.1.2. Dynamic Light Scattering Analysis and Zeta Potential

**Figure 2:**
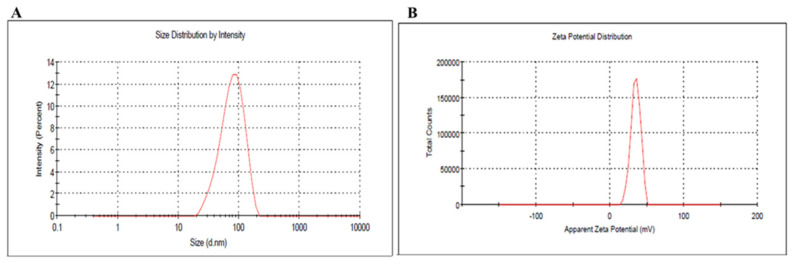
(**A**) Particle size and size distribution (PDI), and (**B**) zeta potential of the developed Au-NP.

The developed Au-NPs were evaluated for their particle size and PDI and the results were represented in Figure 2. The particle size was found to be 82.5 nm with a PDI value of 0.323. On the other hand, zeta potential is regarded as an essential parameter in NPs characterization since the surface charges provide an indication of the stability of the preparation. As displayed in Figure 2B, the zeta potential of the developed Au-NP was 34.8 ± 0.14 mV. This was in accordance with Guo et al, where the prepared Au-NPs using CTAB showed positive zeta potential ranging from +33 to +49 mV [45].

#### 3.1.3. Transmission Electron Microscopy (TEM)

**Figure 3:**
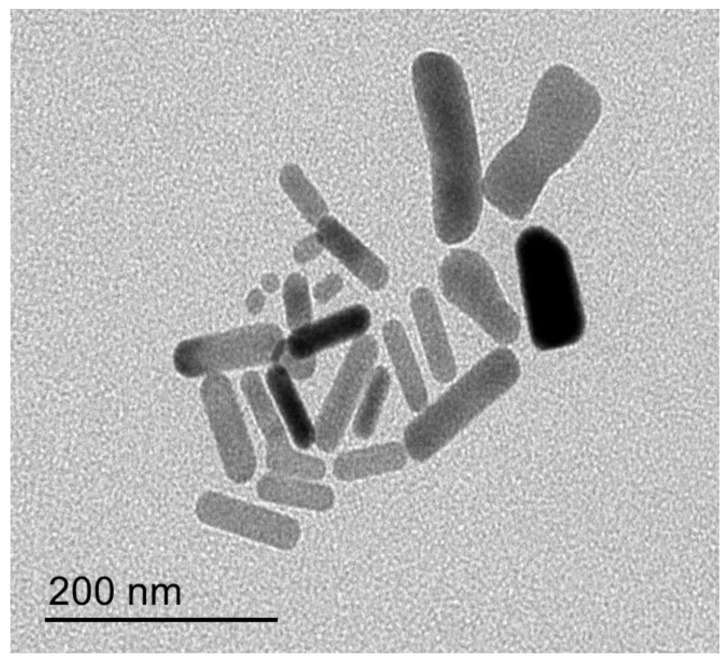
TEM image showing the morphology of rod Au-NP.

Morphology of the prepared Au-NP was evaluated using TEM and the image was obtained in Figure 3. This was in agreement with Zhao et al., who stated that the morphology of the synthesized NPs were long rod nanoparticles [46].

### 3.2. FTIR

**Figure 4:**
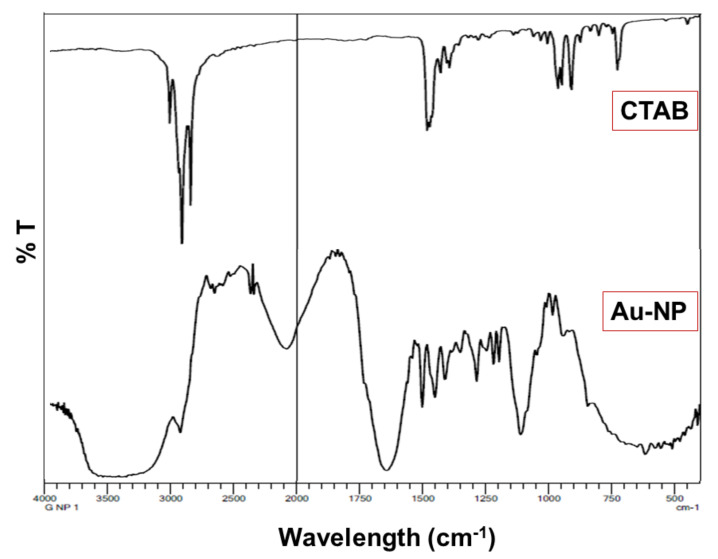
FTIR spectrum of the developed Au-NPs.

Analysis of FTIR of the formulated Au-NPs was determined and the spectrum was displayed in Figure 4. FTIR spectra of the formulated Au-NPs were determined and the spectrum was displayed in Figure 4. FTIR spectra of CTAB showed peaks before 3000 cm^−1^ for different aliphatic C-H stretching vibrations of CH_3_ and CH_2_ groups. In addition to bands around 1650 and 1460 cm^−1^ for N+-C stretching vibrations. Regarding IR spectrum of HAuCl4, it exhibited a broad band in at 330 cm^−1^ at fingerprint region, and in solution and hydrated form showed a broad band at 3300 cm^−1^ due to the OH group of water molecules. On the other hand, NaBH_4_ was characterized at spectrum by 2 B-H Stretching absorption bands around 2100 and 2200 cm^−1^ and its B-H bending mode around 1100 cm^−1^. For ascorbic acid IR spectra, it was confirmed by broad peaks around 3300 cm^−1^ of many hydroxyl groups, The C=O of lactone structure at 1710 cm^−1^, the stretching vibration of C=C at 1674 cm^−1^, The stretching vibration of enol OH group observed at 1322 cm^−1^ and the peak of C-O observed at 1500 cm^−1^. However, AgNO_3_ showed 2 symmetric vibrational bands around 1300 and at ≈ 700–800 cm^−1^ for (N=O) of NO_3_ that was confirmed by 1550–1500 cm^−1^ stretching of the N-O bond [47].

### 3.3. Characterization of the Developed Au-NPs Gel Preparation

#### 3.3.1. Visual Examination

The prepared Au-NP gel was examined visually for the final appearance and was pink coloured, smooth, and homogenous gel.

#### 3.3.2. pH Measurement

The pH of the formulated gel was measured and reported to be 6.67 ± 0.13, which looked to be in great close to the pH of the human skin that assures its safety.

#### 3.3.3. Viscosity Measurement

The viscosity of Au-NP gel was evaluated to be 11450 ± 1212 cP, which is presumed to be in the acceptable range for topical formulations and would not run off easily upon skin application.

#### 3.3.4. Spreadability Determination

Spreadability was determined to evaluate the easiness of the formulation to be spread over the affected area. It was measured to be 57.56 ± 1.16 mm, which is adequate for any topical preparation

### 3.4. Antibacterial Studies

#### 3.4.1. Determination of the Antibacterial and Antifungal Effects 

**Figure 5:**
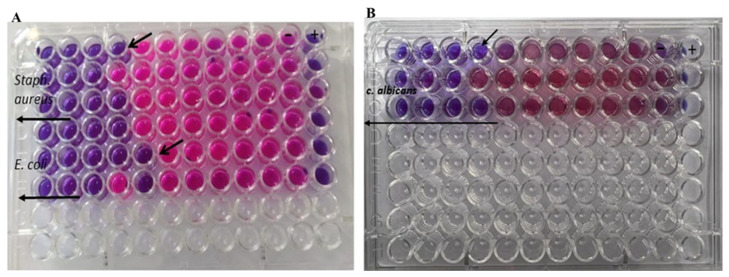
Representative MIC using resazurin dye for (**A**) *S. aureus* and *E. coli* and (**B**) for *C. albicans*.

The rod-shaped nanoparticles that have been developed showed powerful bactericidal and fungicidal activity against a wide range of microbial infections. The antimicrobial activity of rod-shaped gold nanoparticles was determined using a micro broth dilution assay against tested bacterial and fungal strains (Table 1 and Figure 5). The lowest concentration with no apparent development of test pathogens was recorded as the MIC. A bulk of the microbial population is destroyed at concentrations of 0.25–0.125 ng mL^−1^, as determined by MIC/MFC assays (Figure 5). As established for *S. aureus*, *E. coli*, and *C. albicans* isolates using the micro broth dilution method, a relatively low dose of (GNR), ranging from 0.125 to 0.1 ng mL^−1^, should be regarded as bactericidal and fungicidal. Mahmoud et al supported our finding since their study revealed that gold nanorods could be a promising nanoformula having an antibacterial effect against skin disorders [16].

#### 3.4.2. Antimicrobial and Antibiofilm Potential as Characterized by SEM

**Figure 6:**
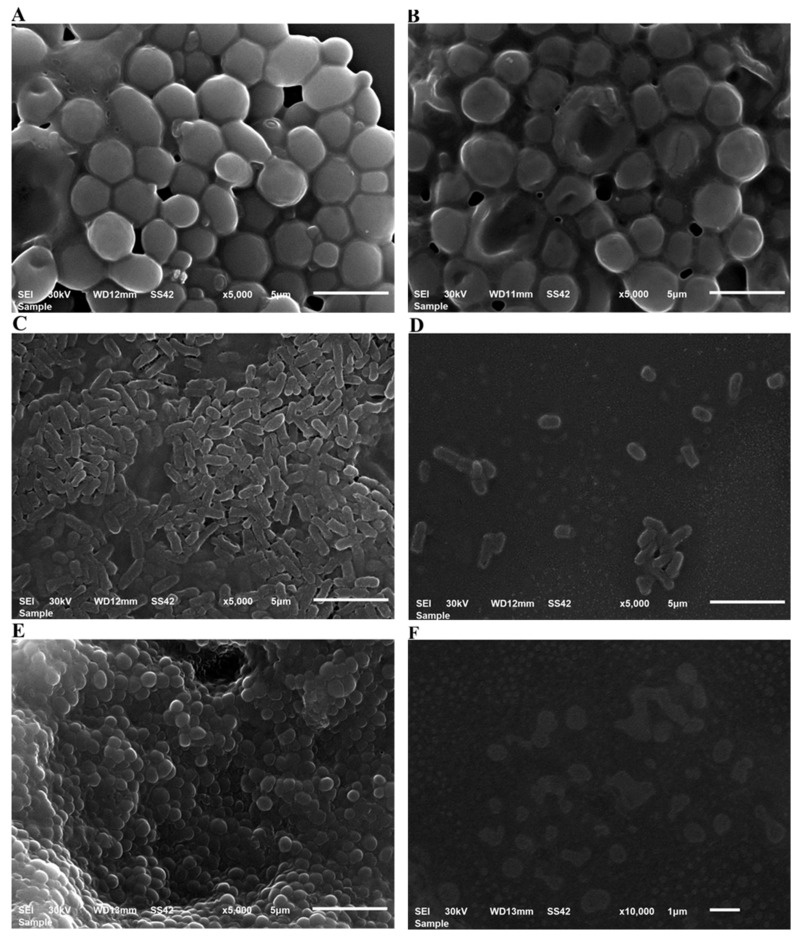
SEM analysis of biofilm structure. SEM images of the biofilms formed on the glass coverslips after 24 h of incubation. (**A**) Control *C. albicans*; (**B**), *C. albicans*. treated with Au-NPs, (**C**) Control *E-coli*; (**D**), *E-coli* treated with Au-NPs (**E**) Control *S. aureus*; (**F**) *S. aureus* treated with Au-NPs.

Nanoparticles’ antibacterial and antibiofilm properties were revealed by SEM as seen in Figure 6. SEM image of bacterial cells after treatment with Au-NPs, demonstrating a reduction in cell number, this is very clear with *S. aureus* and *E. coli*. The control sample had more cells adhered to the surface. Our findings were in accordance with Castillo-Martínez et al., who stated that treatment with gold nanorods resulted in changes in the volume and morphology of the bacteria when compared to the control group without treatment [48].

### 3.5. Assessment of Au-NPs on Excision Wound Healing Parameters in Diabetic Animals 

**Figure 7:**
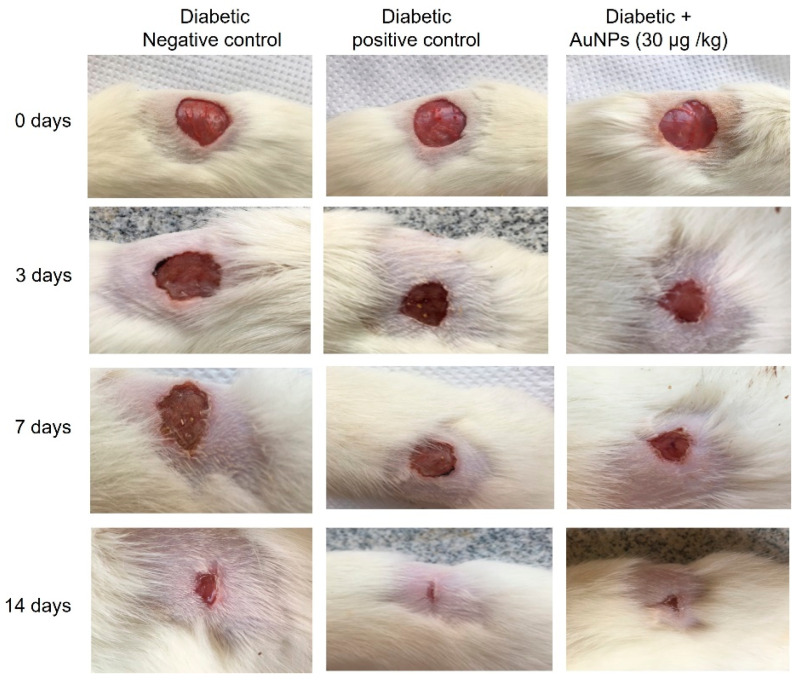
Photographic illustration showing the effect of topical application of AuNPs on wound healing on different days subsequent to the excision wound establishment in diabetic animals.

**Figure 8:**
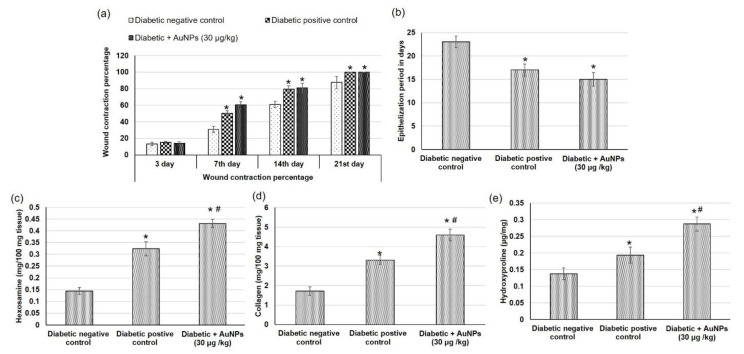
Effect of topical application of Au-NPs in excision wound model on wound healing parameters including (**a**) wound contraction percentage obtained from different experimental groups at 0, 3, 7, 14, and 21 days of post wounding (**b**) epithelialization period, (**c**) hexosamine, (**d**) collagen and (**e**) hydroxyproline contents in diabetic animals. All values were expressed as mean ± SD (*n* = 6). * indicates statistically significant from the diabetic negative control group (*p* < 0.05), # indicates statistically significant from diabetic positive control group using one-way ANOVA followed by Tukey’s test as a post hoc analysis.

**Figure 9:**
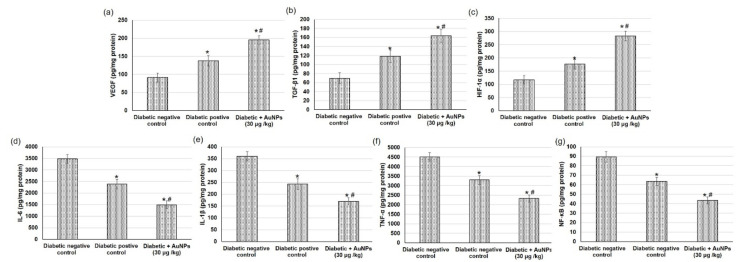
Effect of topical application of AuNPs in excision wound model on angiogenesis-related factors including (**a**) VEGF, (**b**) TGF-β1, and (**c**) HIF-1α and on inflammatory mediators including (**d**) IL-6, (**e**) IL-1β, (**f**) TNF- and (**g**) NF-κB in diabetic animals. All values were expressed as mean ± SD (*n* = 6). * indicates statistically significant from the diabetic negative control group (*p* < 0.05), # indicates statistically significant from diabetic positive control group using one-way ANOVA followed by Tukey’s test as a post hoc analysis.

Figure 7 shows pictures of the wound area taken on different days for the experimental groups to demonstrate the progress of the wound healing ability of Au-NPs in diabetic animals. On the first day after the wound establishment, a bright red color was observed indicating that the recovering of the blood supply to the beneath muscle following the skin injury. On the seventh day subsequent to the wound establishment, a dark brown color was spotted in Au-NPs-treated animals and diabetic positive control, suggesting scab formation, whereas the diabetic negative wounds were still faintly red and inflamed. On the 14th day, Au-NPs-treated and positive control wounds exhibited a significantly reduced wound size when related to the untreated diabetic negative group as revealed in Figure 8. The percentage of wound contraction of excision wounds was statistically (*p* < 0.05) increased in the diabetic rats treated with Au-NPs when correlated to the diabetic negative control group. However, there was no significant difference between Au-NPs and positive control animals in the excision wound contraction percentage (Figure 9a). Regarding the epithelialization time, the mean days of epithelialization increased in the diabetic negative control group, whereas it decreased in the Au-NPs-treated and diabetic positive groups with no significant differences between the treatment groups (Figure 9b).

Hydroxyproline is the main component of collagen which is the main extracellular protein in the skin tissue, making hydroxyproline as an excellent indicator of the collagen content within the skin tissue [49]. This is because any alteration in hydroxyproline amount can indicate any variation in collagen synthesis, reflecting the wound healing process in the injured tissues. The diabetic negative control animals demonstrated decreased hexosamine, collagen, and hydroxyproline contents as demonstrated in Figure 9c–e respectively. On the other hand, topical application of Au-NPs caused significantly increased levels of hydroxyproline, collagen, and hexosamine levels demonstrating enhanced healing processes, compared to both diabetic negative and positive controls.

### 3.6. Assessment of AuNPs on Angiogenesis-Related Factors in Excision Wound Healing in Diabetic Animals 

The angiogenesis process during wound repair performs a dual role of providing the nutrients necessary for the healing tissue and serving for structural repair via granulation tissue construction [50]. The outcomes from the existing investigation showed that the angiogenesis-related factors including HIF-1α, TGF-β1, and VEGF contents in wound tissues were inferior in the negative diabetic group to the positive diabetic group. Furthermore, topical application of AuNPs displayed a significant intensification in VEGF, TGF-β1, and HIF-1α levels compared with diabetic positive animals (Figure 9a–c).

### 3.7. Assessment of Au-NPs on the Inflammatory Mediators in Excision Wound Healing in Diabetic Animals 

In the current study, we evaluated different inflammatory mediators to specify the inflammation status happening during the diabetic wound healing process. Diabetic non-treated animals revealed significant escalation in numerous inflammatory mediators such as IL-6, IL-1β, TNF-α, and NF-κB when linked to positive diabetic animals. While, topical application of AuNPs demonstrated diminished inflammation, as verified by the significantly inferior level of inflammatory mediators when compared to diabetic positive animals (Figure 9d–g).

### 3.8. Assessment of AuNPs Antioxidant Activity and Lipid Peroxidation in Excision Wound Healing Parameters in Diabetic Animals 

**Figure 10:**
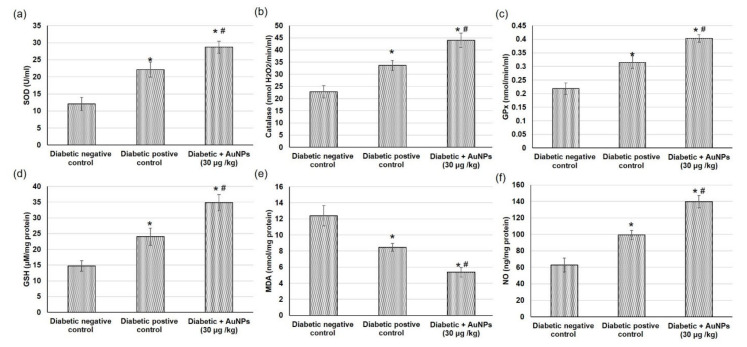
Effect of topical application of AuNPs in excision wound model on antioxidant enzymes activities including (**a**) superoxide dismutase (SOD), (**b**) catalase (CAT), and (**c**) glutathione peroxidase (GPx), (**d**) glutathione (GSH) content, (**e**) lipid peroxidation expressed as malondialdehyde (MDA) and on (**f**) nitric oxide (NO) content. All values were expressed as mean ± SD (*n* = 6). * indicates statistically significant from the diabetic negative control group (*p* < 0.05), # indicates statistically significant from diabetic positive control group using one-way ANOVA followed by Tukey’s test as a post hoc analysis.

Oxidative stress persistence during the wound healing process is detrimental, specifically in diabetic wounds. Figure 10 illustrates the outcomes of the topical application of Au-NPs on different antioxidant enzymes including CAT, SOD, GPx, on the GSH content, and on lipid peroxidation expressed as malondialdehyde (MDA), and finally on the NO content on the excision wound healing process. Diabetic non-treated animals exhibited a significant reduction in antioxidant enzyme activity including CAT, SOD, and GPx and GSH content and NO levels as well as augmented lipid peroxidation. 

On the other hand, animals treated with AAu-NPs presented a significant intensification in antioxidant enzymes (CAT, SOD, and GPx) and GSH content in, as compared to diabetic-positive animals. This action could be attributed to the reduction in reactive oxygen species (ROS) manufactured with Au-NPs treatment. In addition, a distinguished reduction in MDA level (lipid peroxidation marker) in the wound tissue obtained from Au-NPs treated animals when compared to the positive control animals, indicating that the topical application of Au-NPs reduced the secondary oxidation product content. Additionally, Au-NPs significantly amplified the nitric oxide level when compared to diabetic positive control rats.

## 4. Discussion

### 4.1. Characterization of Au-NPs

As per UV–Visible spectroscopic determination, it was performed and showed a peak at 525 nm, which is the characteristic absorption peak for the formula. Regarding particle size and size distribution, the developed Au-NP exhibited a particle size of 82.5 nm with a corresponding PDI value of 0.323, which indicates that the distribution of the particle size falls within a narrow range of sizes. It was previously reported that PDI values lower than 0.7 point toward homogeneity of the particle size and denoted an ideal formulation [51]. However, the zeta potential was found to be 34.8 ± 0.14 mV. It is highly obvious that Au-NP tends to carry a characteristic positive charge, which is certainly ascribed to the presence of CTAB that could induce a positive charge since it is a positively charged surfactant [52]. Additionally, the value of zeta potential emphasized the stability of the formulation since it was reported that particles are usually considered stable when showing zeta potentials more positive than +30 mV or more negative than −30 mV [53]. The image exhibited the rod shape of the particles in addition to emphasizing the particle size obtained from the zeta sizer with a diameter less than 100 nm. Concerning morphology evaluation, the image obtained from the TEM study exhibited the rod shape of the particles in addition to emphasizing the particle size obtained from the zeta sizer with a diameter less than 100 nm.

### 4.2. FTIR Study

FTIR study was implemented and it was clearly apparent that the characteristic peaks of all excipients were present and being distinguished in the spectrum. However, in the case of the IR spectrum of HAuCl4, it was noticed that the characteristic peaks of gold were not apparent indicating the reduction by ascorbic acid although the fingerprint region of Au is still present signifying the existence of gold.

### 4.3. Characterization of the Developed Au-NPs Gel Preparation

The prepared Au-NP gel was evaluated for all the parameters to check its suitability for topical use. All the evaluated parameters were in the acceptable range with regard to the topical formulation.

### 4.4. Antibacterial Studies

Nanotechnology has gotten a lot of attention since nanoparticles have different properties than their bulk counterparts. Human pathogenic bacteria such as *E. coli* and *S. aureus* have been used to test nanoparticle antibacterial properties [4]. In microbiology, the MIC and MBC values are the most important tests for determining an agent’s antibacterial activity. The MIC is the lowest concentration of an antimicrobial agent that prevents observable bacterial growth (bacteriostatic activity), whereas the MBC is the highest concentration that causes microbial mortality (at which it is bactericidal) [54]. MIC and MBC data showed that Au-NPs had bacteriostatic and bactericidal properties at concentrations of 0.25–0.125 ng mL^−1^ against *S. aureus*, *E. coli*, and *C.albicans*, respectively.

Because of the Au-NPs synthesis method or cell growth determination method, there are no significant variations in the antibacterial impact of Au-NPs on Gram-negative and Gram-positive bacteria, besides antifungal activity which is in agreement with Zhang et al’s findings [55].

Metal NPs’ antibacterial effect has been extensively researched, although their specific mechanisms of action are still unknown. The antibacterial effect of gold nanoparticles has been attributed to a number of processes. The nanoparticles attach tightly to the microbes’ surfaces and puncture the cell wall. This causes cell contents to flow out, resulting in the death of bacterial cells [56]. Reactive oxygen species generation, cation release, biomolecule destruction, ATP depletion, and membrane contact are all other factors that may contribute to the death of bacterial cells [57,58]. It is also possible that the nanoparticle can interact with the phosphorus or sulphur moieties of DNA, which may hinder the DNA replication and subsequently cause the death of bacteria [59]. GNPs can stimulate protein refolding via interactions between unfolded protein and oppositely charged AuNPs, depending on their cytotoxicity on the surface [60]. Furthermore, it has been suggested that the surface charge of nanoparticles has a role in their cytotoxicity [61]. These findings were in accordance with Zhou et al., 2012, [62] who found that biophysical interactions between NPs and bacteria may occur via biosorption, aggregation, and cellular absorption, resulting in membrane degradation and toxicity. However, in order to improve the efficacy of NPs in disease treatment, a thorough understanding of mechanisms of antibacterial activity is required.

The bactericidal action of Au-NPs, according to Zhang et al. [55], may be owing to the bactericidal activity of co-existing molecules that are not entirely eliminated from AuNPs, such as gold ions, surface coating agents, and chemicals used in the synthesis. The coating composition has a significant impact on Au-NP characteristics [62].

Nanoparticles’ bactericidal effects were also stronger for Gram-negative bacteria than for Gram-positive bacteria. This is due to a change in the cell wall’s composition. Gram-positive bacteria have much rigid cell wall network with a thick layer of peptidoglycan that makes them to resist mechanical rupture. However, Gram-negative bacteria possess a one-molecule thick cell membrane network [63,64]. These factors would probably enhance the activity of NP against Gram-negative bacteria.

### 4.5. In Vivo Evaluation of the Wound-Healing Activity

The in vivo wound-healing ability was conducted through determining wound area reduction, tissue re-epithelialization, collagen deposition, angiogenesis, inflammatory mediators, and antioxidant enzyme activities. The wound healing process happens in four different coinciding phases, which are hemostasis, inflammation, proliferation, and remodeling [65]. During the proliferative phase, remarkable amounts of cells including fibroblast, keratinocytes, and endothelial cells transfer to the wounded area. The extracellular matrix (ECM) including proteoglycans, hyaluronic acid, collagen, and elastin accumulates to form a new granulation tissue to substitute the original. Numerous kinds of cytokines and growth factors participate in the wound healing process including the transforming growth factor- β family (TGF-β), interleukin (IL) family, and angiogenesis factors (i.e., vascular epidermal growth factor VEGF [66].

In the current study, topical application of Au-NPs caused a significantly increased percentage wound reduction, lesser time for epithelialization, and augmented hydroxyproline, collagen, and hexosamine levels demonstrating improved healing processes. Similarly, Raghuwanshi, et al. [67] showed that gold nanoparticles biosynthesized using Woodfordia fruticosa which were complex with carbopol demonstrated a higher level of hydroxyproline and collagen fibers causing better tensile strength as well as an accelerated healing process. Furthermore, gold nanoparticles exhibit a positive impact on the healing of wound infections by means of photobiomodulation therapy [20].

Furthermore, the current study showed that topical application of Au-NPs displayed a significant intensification in angiogenesis-related factors such as VEGF, TGF-β1, and HIF-1α levels. Furthermore, Au-NPs demonstrated diminished inflammation, as verified by the significantly inferior level of inflammatory mediators IL-6, IL-1β, TNF-α, and NF-κB. Regarding antioxidant activity, Au-NPs displayed potent antioxidant activity via intensifying the activity of antioxidant enzymes including CAT, SOD, and GPx, GSH, and NO contents, and on diminishing lipid peroxidation in the excision wound healing process.

Lau, Bidin, Islam, Shukri, Zakaria, Musa and Krishnan [20] argued that the anti-inflammatory response and effective angiogenesis were the potential cause of effective wound repair obtained by Au-NPs alone and with photobiomodulation. In another study, the wound healing potential of Au-NPs was attributed to its antioxidation, anti-inflammation, and non-antiangiogenesis properties which lead to the formation of fibroblasts and decrease in the apoptosis of cells eventually contributing to the wound healing process [23]. The antimicrobial and antioxidative effects of gold nanoparticles proved very effective in wound healing and regeneration of damaged collagen tissues [49]. Furthermore, gold nanoparticles are involved in the secretion of proteins (IL-8, IL-12, VEGF, and TNF-α) which are important candidates for wound healing via their antiangiogenic and anti-inflammatory activity [68]. Also, gold nanoparticles, together with epigallocatechin gallate and α-lipoic acid, demonstrated to have high antioxidant and anti-inflammatory activities causing skin wound healing [24].

## 5. Conclusions

In the present investigation, nanotechnology was exploited via developing gold nanoparticles using the reduction method. The nanoparticles were characterized for their particle size, zeta potential, morphology, and FTIR. They were investigated for their antibacterial activity and efficiency for wound healing in diabetic animals. The nanoparticles provide proper particle size, zeta potential, and rod-shaped particles. They could provide efficient antibacterial behavior against different bacterial strains. Ultimately, gold nanoparticles exhibited promising wound healing activity in STZ induced diabetic animals.

## Figures and Tables

**Table 1:** MIC and MBC values of Au-NPs suspensions against different microbial strains.

Strains	MIC/MBC
*Staphy. aureus*	0.25/0.1 nmole/mL
*E. coli*	0.125/0.125 nmole/mL
*C. albicans*	0.25/0.5 nmole/mL

## Data Availability

Not applicable.
