# Correction: Soliman et al. Evaluating Antimicrobial Activity and Wound Healing Effect of Rod-Shaped Nanoparticles. *Polymers* 2022, *14*, 2637

**DOI:** 10.3390/polym17020183

**Published:** 2025-01-14

**Authors:** Wafaa E. Soliman, Heba S. Elsewedy, Nancy S. Younis, Pottathil Shinu, Lamis E. Elsawy, Heba A. Ramadan

**Affiliations:** 1 Department of Biomedical Sciences, College of Clinical Pharmacy, King Faisal University, Alhofuf 36362, Al-Ahsa, Saudi Arabia; spottathail@kfu.edu.sa; 2 Department of Microbiology and Immunology, Faculty of Pharmacy, Delta University for Science and Technology, Mansoura 11152, Egypt; lamis.elbaz@deltauniv.edu.sa (L.E.E.); heba.musa@deltauniv.edu.eg (H.A.R.); 3 Department of Pharmaceutical Sciences, College of Clinical Pharmacy, King Faisal University, Alhofuf 36362, Al-Ahsa, Saudi Arabia; helsewedy@kfu.edu.sa (H.S.E.); nyounis@kfu.edu.sa (N.S.Y.)

In the original publication, there was a mistake in Figure 7 as published [1]. The images of wounds were belonging to the same animal taken at different times. Therefore, we changed the negative control one in Figure 7. The corrected Figure 7 appears below. The authors state that the scientific conclusions are unaffected. This correction was approved by the Academic Editor. The original publication has also been updated.

## Figures and Tables

**Figure 7:**
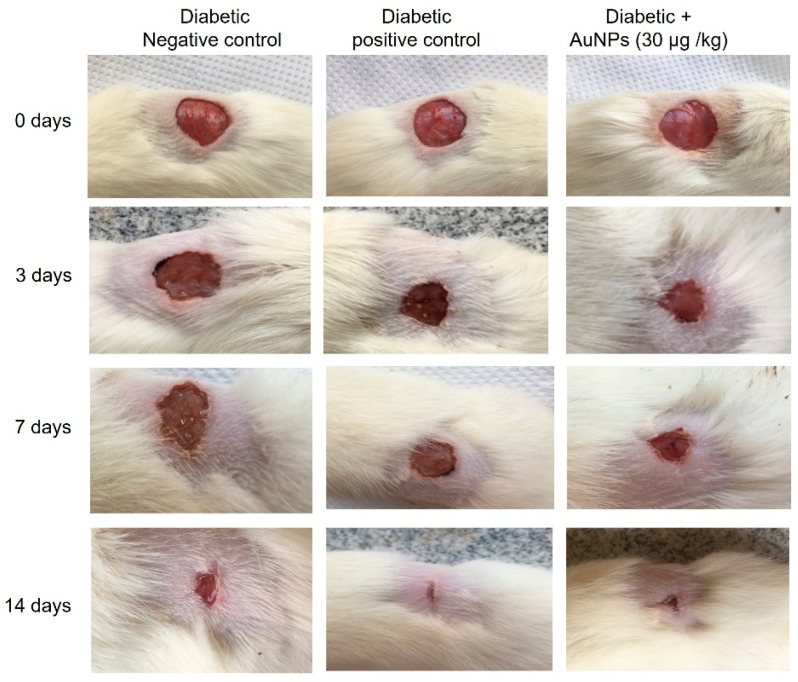
Photographic illustration showing the effect of topical application of AuNPs on wound healing on different days subsequent to the excision wound establishment in diabetic animals.
